# Upstream solutions for price-gouging on critical generic medicines

**DOI:** 10.1186/s40545-016-0064-8

**Published:** 2016-05-02

**Authors:** Adam R. Houston, Reed F. Beall, Amir Attaran

**Affiliations:** University of Ottawa, Room 221 1 Stewart St, Ottawa, ON K1N 6N5 Canada

**Keywords:** Essential medicines, Generics trolling, Cycloserine, Pyrimethamine, Pharmaceutical pricing, Generic drugs

## Abstract

Exorbitant price increases for critical off-patent medicines have received considerable media attention in recent months, leading to an investigation by the U.S. Senate. However, much of this attention has focused upon the companies that initiated the price increases, all of whom had recently acquired the drugs in question. Overlooked are upstream interventions with the originators of these drugs to prevent generics trolling in the first place. Using the particular example of Eli Lilly and Company’s efforts to divest itself of cycloserine, a flawed process that paved the way for the recent price hike by Rodelis Therapeutics, this article highlights the responsibilities of drug originators, and safeguards to ensure similar rights transfers do not affect ongoing affordable access.

## Correspondence/findings

In the fall of 2015, to the surprise and dismay of clinicians and their patients, the North American price of cycloserine—a WHO Essential Medicine for multidrug-resistant tuberculosis (MDR-TB)—increased from about $500 to $10,800 for thirty 250 mg capsules, or close to $800,000 per patient (assuming 750 mg/day for 2 years) [1].

The cycloserine debacle exemplifies a disturbing new trend of generic drug trolling, where companies acquire the marketing and manufacturing rights for off-patent, critical medicines with a single source (so a *de facto* monopoly) and raise prices astronomically. In this instance, North American rights around cycloserine had just been acquired by Rodelis Therapeutics. Around the same time, Turing Pharmaceuticals obtained similar North American rights for pyrimethamine, another WHO Essential Medicine, and jacked up the price by more than 5500 % overnight, in the process creating the quintessential poster boy for pharmaceutical company greed in then-CEO Martin Shkreli [2].

This unscrupulous rent-seeking—with none of these drugs have the trolling companies offered any new innovation other than in their pricing strategies—has drawn considerable media and political attention. Not only has generics trolling helped fuel a broader conversation about pharmaceutical pricing in the United States as part of the current U.S. presidential campaign, the companies above were called to testify before a U.S. Senate Commission [3]. Even more importantly, numerous policy suggestions have emerged, ranging from simplifying FDA processes to promote market entry for generic competitors, to tackling anticompetitive behaviors used to impede competition, to easing restrictions on importation of foreign sources [4].

While these suggestions may all contribute to improving pharmaceutical access and pricing, we suggest that an important complementary strategy is to address the upstream conditions that have allowed generics trolling to occur in the first place. These conditions arise from the irresponsible divestment of critical drugs by the original innovators.

The case of cycloserine illustrates how this can occur even under the guise of a philanthropic effort to promote access. Through Eli Lilly and Company’s MDR-TB Partnership, which Lilly describes in a report as “the largest philanthropic undertaking in the company’s history” [5], Lilly offloaded two unprofitable drugs—cycloserine and capreomycin—onto seven generics companies in different territories by transferring manufacturing know-how, marketing rights, and other intellectual property (e.g. cycloserine’s trademark, Seromycin®). In North America, Lilly completely divested exclusive rights to cycloserine to Purdue University’s Chao Center. This 2007 transfer made Chao the only North American supplier until August 2015, when Chao unexpectedly re-transferred cycloserine to Rodelis. Rodelis promptly hiked its price twenty-fold. Protests ensued, by us and others, until Chao announced on September 21, 2015 that it had regained the rights to cycloserine and would reduce the price to $1050—a bittersweet outcome, given this is still double their original price from the month before [6].

A similar pattern—divestment, multiple changes in ownership, and ultimately massive price-hikes after being acquired by trolls—occurred for GlaxoSmithKline’s (GSK) pyrimethamine (Daraprim®), although GSK’s divestment held no pretense of philanthropy. Yet while Andrew Witty, CEO of GSK, criticized Turing’s price increase, he failed to acknowledge that it was GSK’s divestment from this drug that allowed Turing to acquire it in the first place [7]. Ultimately, these divestments have taken place with insufficient measures in place to ensure continued, affordable access. They impose no price ceilings or other conditions for the future, but instead relinquish control over pricing and access decisions, freeing the successor companies to price-gouge at will.

In 2001, the average price Lilly charged for cycloserine in high-income countries was $3.38 per capsule—a very far cry from the $35 they cost from the Chao Center, let alone the $360 that Rodelis briefly charged in North America [8]. Pity the unfortunate patients in the Canadian and U.S. markets, for whom “the largest philanthropic undertaking in [Lilly’s] history” has resulted in prices that seem decidedly misanthropic. Lilly does not deny this problem, and writes, in the same report noted above, “that current prices for MDR-TB medicines are now, in some cases, higher than they were when Lilly’s was subsidizing the price. However, while prices have not fallen in all cases for patients, the cost of production has fallen as a result of technology transfer.” In short, patients might pay more, but it is costing the manufacturers less.

We asked Lilly why it gave the successor companies unlimited discretion over cycloserine pricing. In responses to us and the *New York Times*, Lilly answered that antitrust rules prohibited it having downstream control over cycloserine’s price. It is not clear what rule Lilly is referring to, particularly given that common sense also opposes this idea: Lilly’s decision to stop selling and earning revenue off cycloserine made it a mere bystander in that market, and therefore unlikely to be guilty of criminally rigging the market (in favor of consumers, no less).

We believe that Lilly and other drug companies that choose to relieve themselves of manufacturing responsibilities for an important medicine need to do so in an ethical and responsible manner. Responsibility to one’s invention and to public health each require companies to plan thoughtfully for what will happen to their product after they no longer manufacture it. Indeed, the decision to stop manufacturing a crucial drug, even one serving only a small market, should itself be carefully considered. It is certainly not unreasonable to expect that corporate good citizenry should encompass continuing to ensure access to vital legacy drugs serving a critical market. In an era where “Big Pharma” is increasingly used as a pejorative, it also makes good sense for drug originators to embrace, rather than discard, important legacy drugs. Highlighting a product worthy of being deemed an “essential medicine” is something that could only improve public perceptions—provided it comes with commitments to affordable, ongoing access.

Should a company wish to license other manufacturers to lighten the financial burden of serving the market, that is entirely acceptable, but it is our view that originator suppliers remain ethically bound to ensure a stable, safe, and adequate supply. If they choose to divest, companies should at the very least include some form of enduring price ceiling (there are many options) in any agreement that transfers marketing rights to a generics company, lest that successor, or as has frequently been the case the successor’s successors, abuse their discretion to price-gouge patients. It also makes good sense to be mindful of the size of relevant markets when dividing such rights between multiple parties internationally to help ensure the sustainability of the transfer; it seems obvious that carving off a tiny market—like North America—to a manufacturer without any track record and few if any other products in its portfolio would almost inevitably have to result in higher prices. Indeed, this is why merely calling for increased domestic competition is an overly simplistic approach. The vulnerability that generics trolls exploit is that the previous manufacturer is seeking to disavow themselves of production responsibilities precisely because it is not sufficiently profitable, even as a sole-supplier; in the above cases, this has been true not only for the originator but also for the initial successor rights-holder. The market for these drugs is frequently too small to sustain multiple suppliers or promote competition at the national or regional level.

Thus, maintaining access to important legacy drugs serving crucial patient markets, not simply the bottom line, should form a part of corporate responsibility and ethics. Nevertheless, circumstances may still warrant some degree of market intervention by governments, even before a troll has taken advantage of the situation. Drug manufacturers in the United States are currently required to notify the government of the discontinuance or interruption in the production of life-saving drugs [9]. Foreign importation has been permitted in response to such shortages. Such a principle, including foreign importation when necessary, could be expanded to ensure that any divestment of a critical drug not have a detrimental effect on access, both physical and financial. There is precedent in other fields for protecting critical access during transfer; for instance, the transfer of airport slots between airlines requires the determination “that the transfer will not be injurious to the essential air service program” [10]. This may also be a more politically palatable solution than opening the broader pharmaceutical market to foreign suppliers, although that approach also offers benefits worth considering.

The current state of affairs is such that the system remains vulnerable to unscrupulous trolls preying upon small markets served by a sole-source supplier. This essentially inverts the common stereotype of what makes pharmaceuticals unaffordable, as drugs that were affordable under their originators explode in price on the generic market. At the root of the problem are drug originators like Lilly and GSK, who sully their past innovations through irresponsibly off-loading manufacturing responsibilities. They are also the best positioned to prevent similar occurrences in the future. While measures like addressing obstacles to increased generic competition have an important role in lowering drug prices, they are not a complete solution, particularly for tiny, vulnerable markets. Preventing the problem through more responsible and ethical divestment practices is preferable to treating it after it occurs.
